# Quality of life in assisted living facilities for seniors: A descriptive exploratory study

**DOI:** 10.1002/nop2.2084

**Published:** 2024-03-01

**Authors:** Elham Alomari, Claudia Steinke

**Affiliations:** ^1^ Nursing Faculty, Faculty of Nursing University of Calgary in Qatar Doha Qatar; ^2^ Faculty of Health Sciences University of Lethbridge Lethbridge Alberta Canada

**Keywords:** ageing, assisted living, quality of life, seniors

## Abstract

**Introduction:**

Promoting individuals' health across different life spans has always been key to a holistic nursing practice. Seniors are a diverse population who go through many physical and mental changes as they age. During the last decade, assisted living facilities (ALFs) have dramatically increased in numbers to provide care and living services in a home‐like environment.

**Aim:**

The aim of this descriptive exploratory study was to explore the quality of life as perceived by seniors who reside in assisted living facilities (ALFs).

**Design:**

This study utilized a descriptive exploratory design to investigate the quality of life of seniors living in ALFs.

**Methods:**

Seventeen residents from two ALFs were interviewed to gather their perspectives on the quality of their lives while living in an ALF. The interviews were conducted by the researcher and were audio‐recorded and transcribed verbatim. The data were analysed using thematic analysis.

**Results:**

Three major themes surfaced from residents' descriptions: ‘physical environment’, ‘social environment’ and ‘home‐like atmosphere’. The quality of life in ALFs was found to be predominantly an outcome of the exchange between the personal capability of residents to adapt to changes and the capacity of the facility to meet residents' diverse needs.

**Patient or Public Contribution:**

Participants who discussed their quality of life in ALFs provided profound insights into this aspect of their lives. The findings from this study can potentially enlighten ALF stakeholders and enhance the quality of life for seniors residing in these facilities.

## 1 INTRODUCTION

Ageing is a global phenomenon with far‐reaching implications for various stakeholders, including policymakers, healthcare professionals, seniors and their families. The global population of individuals aged 65 and above is growing rapidly, projected to reach 1.5 billion by 2050, from 703 million in 2019 (UN, 2019). In Canada, the senior population represents the fastest‐growing segment of society, with projections indicating a rise from 4.2 million to 9.8 million between 2005 and 2036, possibly accounting for over a quarter of the Canadian population by 2056 (Statistics Canada, 2019). With advancing age, individuals often experience increased chronic diseases and physical and cognitive impairments, impacting their ability to live independently (Zheng et al., 2020).

As individuals age, the need for assistance with daily activities and personal care becomes more prevalent. Therefore, there is a growing demand for supportive living options for seniors, particularly those aged 65 and older (Johnson et al., 2018). Assisted living facilities, also known as supportive living options, have emerged to meet this need, offering an alternative for seniors who cannot remain at home but do not require the level of care provided in nursing homes (Dalmer, 2019; Lehning et al., 2017). The mission of assisted living facilities is to give a supportive and home‐like environment for older adults and individuals with disabilities, offering assistance with activities of daily living, personalized care, safety and social engagement, while professionals such as personal support workers, licenced practical nurses, recreation therapists and other support staff work to ensure residents' well‐being and independence (Alberta Health Services, 2012). It has seen rapid growth in the seniors' care market in Canada since the mid‐1990s and currently serves a substantial percentage of Canadian seniors requiring assistance with daily living but not 24‐h medical care (CHSPR, 2012; Government of Alberta, 2014).

Quality of life is considered to be one of the most desirable outcomes in all domains of healthcare. How to best support quality of life among seniors has become a growing health and social policy concern. As stated earlier, definitions of quality of life are numerous and inconsistent because of the multiple perspectives and assessment methods. Measuring quality of life is complex; several tools and scales have been developed to assess quality of life in various contexts such as the World Health Organization Quality of Life (WHOQOL) instrument, EuroQol 5‐Dimension (EQ‐5D), Quality of Life Enjoyment and Satisfaction Questionnaire (Q‐LES‐Q), Long Term Care Quality Of Life assessment scale (LTC‐QOL) and the Satisfaction with Life Scale (SWLS) (Arensberg et al., 2023). The individualistic, subjective and multidimensional nature of the concept of quality of life makes it difficult to assess and measure (Beerens et al., 2013; Borglin et al., 2005). There is also disagreement about the meaning of quality of life in the literature; different terms are used such as ‘well‐being’, ‘life satisfaction’ and ‘health status.’ These various terms serve to highlight the lack of agreement in the literature (Bowling et al., 2002; Howell & Cleary, 2007; Phillips, 2012). The Center of Health Promotion at the University of Toronto has developed a definition of quality of life that is applicable to all individuals from different age groups. According to this center, quality of life refers to the degree to which an individual enjoys the important possibilities of his or her life, these possibilities reflect an interaction between personal and environmental elements (Raphael, 2010).

Despite the growth of seniors' supportive living options, research has primarily focused on organizational and structural aspects, with limited attention given to understanding residents' perceptions of their quality of life in these facilities and factors affecting their physical and psychological well‐being (Burdick et al., 2005; Duan et al., 2020; Hawes et al., 2003; Zimmerman et al., 2003). The concept of quality of life is subjective and multifaceted, and its meaning varies among individuals (Beerens et al., 2013; Borglin et al., 2005). It encompasses various terms, such as well‐being, life satisfaction, and health status, and goes beyond traditional clinical approaches to include qualitative indicators (Parmenter, 1994; Schalock et al., 2016).

In the existing literature, quality of life among seniors is often defined in the context of successful ageing, primarily focusing on the absence of physical and cognitive impairments or reduced activities of daily living (Duan et al., 2020; Howell & Cleary, 2007). Few studies have examined quality of life from the seniors' own perspective (Bowling et al., 2002; Howell & Cleary, 2007). Self‐rated health and well‐being consistently emerge as more influential in determining quality of life than objective socio‐demographic and economic indicators (Berg et al., 2011; Bowling et al., 2002).

This study is important because it addresses the gap in research about the quality of life experienced by seniors in assisted living facilities from their perspective. By understanding how seniors define and perceive their quality of life in these settings, this research can contribute to improving both research and practice in the field of seniors' supportive living.

## 2 METHODOLOGY

### 2.1 Research design

A descriptive qualitative approach was adopted to explore and describe the experience of seniors and allow for an in‐depth understanding of residents' perceptions of quality of life, and the meaning they give to that concept while living in ALFs. Qualitative descriptive inquiry is the method of choice when straight descriptions of phenomena are desired (Bradshaw et al., 2017; Sandelowski, 2000).

### 2.2 Setting

The study was conducted in two assisted living facilities (ALFs) in Alberta, each operated by different organizations. The first ALF operates under a faith‐based health organization, known for its extensive healthcare services, including acute care, continuing care, assisted living, hospice, rehabilitation, respite care, and seniors' housing. This organization is committed to serving individuals of all faiths and backgrounds, emphasizing quality improvement through planning, data analysis, and collaboration among caregivers. The facility accommodates 200 suites, including dementia‐specific areas, promoting resident choice and a home‐like environment.

The second ALF is operated by a private, for‐profit organization, specializing in aging in‐place models. Founded in 1998, their mission is to provide high‐quality personal care in a home‐like setting. This ALF, with 112 suites, offers studio and one‐bedroom options, emphasizing independence, dignity and choice. It features a unique design with a focus on community spaces, including dining areas with warm, inviting atmospheres. Resident rooms are equipped with safety measures, air conditioning, and personal climate control.

### 2.3 Sampling and recruitment

Participants were selected from two distinct assisted living facilities affiliated with separate healthcare organizations. These organizations were motivated to participate in the study to gain insights into the quality of services they offer to their clients. Three facilities were approached, and two of them agreed to be part of the study. Participants were recruited based on convenience and purposive sampling. The main criteria for inclusion are as follows: a participant was a senior (65 years and older), a resident of the facility for a minimum of 6 months, cognitively capable, and willing to participate in the study. Demographic information including age, gender, level of education, marital status, occupation and location of primary residence before residing at the ALF was collected. Recruitment started by means of posters and personal invitation letters for those who met the criteria. Nine participants agreed to participate from the first facility, and eight from the second facility. A total of 17 participants were recruited from the two sites. A written informed consent was discussed with all participants to ensure that they understood the research purpose and process, and all questions were answered and then signatures were obtained. The researcher then conducted in‐depth personal semi‐structured interviews with 17 residents from two ALFs. Ethics approval to conduct the study was obtained from the Organizational Health Research Centre and the Health Research Ethics Board.

### 2.4 Data collection

Semi‐structured interview questions were used, see Appendix 1: Resident Interview Protocol. The research team behind this study was comprised of researchers in the fields of management and health sciences. Their extensive backgrounds and expertise in these disciplines enhance the study's credibility. Importantly, the interviewer had no pre‐existing relationship with any of the interviewees, ensuring impartiality in the data collection process.

All interviews took place inside the facilities in a private room at the convenience of the participant. Each individual interview took approximately 45–60 min, and was audio recorded with the participant's consent; pseudonyms were assigned to participants. Data saturation was achieved when no new themes emerged from subsequent interviews. The point of saturation was recognized when repeated interviews consistently yielded redundant data and no novel insights were obtained. Hennink and Kaiser (2022) propose that saturation typically emerges in the range of the 9th to 17th interviews, a range that aligns with the number of interviews conducted in this research. Each interview was confidentially transcribed verbatim; the researcher reviewed the transcript along with the recorded interview line by line to ensure no discrepancies. All participants validated the transcribed data were correct; no changes were made to any of the reviewed transcripts.

### 2.5 Data analysis

The NVIVO software was utilized for the independent extraction, coding and analysis of data by a single researcher while employing the thematic analysis method developed by Braun & Clarke, 2006. Thematic analysis is a method for identifying, analysing and reporting patterns or themes in the data. It organizes and describes the data set in rich detail. The following steps, as proposed by Braun and Clarke (2006), were adopted in this study:

1. Getting familiar with the data by reading the transcripts several times. During this phase, the researcher started taking notes, searching for patterns and meanings and marking ideas for initial coding.
2. Generating initial codes: Codes refer to ‘the most basic element of the data or information that can be assessed in a meaningful way regarding the phenomenon’ (Braun & Clarke, 2006, p. 88). In carrying out the initial coding, the researcher named ‘chunks’ of data with initial code that categorized and summarized each piece of data. Qualitative data analysis software (QDA Miner) was used to organize data.
3. Searching for themes: By ordering codes into categories, all data relevant to potential themes were gathered.
4. Reviewing themes: During this phase, formulated themes were refined and reviewed.
5. Defining and naming themes: A satisfactory thematic map was made from the analysed data.
6. Producing the report: Rich and compelling examples were extracted from the analysed data. The final analysis of selected extracts was assured by relating the analysis back to the research question and literature, producing a rich and scholarly report of the analysis.

### 2.6 Description of participants

A total of 17 participants were recruited: 13 female (76.5%) and four male (23.5%) seniors from two ALFs. All participants were Caucasian with a mean age of 84 years (range = 69–93). Eight participants (47%) were widowed while seven participants (41%) were married and two participants (12%) were divorced. Thirteen participants (76%) lived in their own homes before moving to ALF and one participant (6%) moved from long‐term care, while two participants (12%) moved from other ALFs and one participant (6%) came from a rental retirement community. For all participants, the average length of stay in the ALF was 30 months at the time of interviews (range = 6–120 months). Two participants (12%) were residents for more than 60 months (5 years). Further descriptions of demographic data are provided below (Table 1).

**TABLE 1:** Demographic Data Descriptions (*n* = 17).

Characteristic	Number (percentage)
Gender	
Female	13 (76.5%)
Male	4 (23.5%)
Age range (years)	
65–75	4 (24%)
76–85	5 (29%)
>85	8 (47%)
Race	
Caucasian	17 (100%)
Marital status	
Married	7 (41%)
Widowed	8 (47%)
Divorced	2 (12%)
Education	
Bachelor	1 (06%)
Diploma	7 (41%)
High school	5 (29%)
Middle school	4 (24%)
Occupation before retirement	
Professional	6 (35%)
Clerical/service	8 (47%)
Unemployed	3 (18%)
Residence before moving to ALF	
Own home	13 (76%)
Long‐term facility	1 (06%)
ALF	2 (12%)
Retirement community	1 (06%)
Length of stay at ALF range (6–120) months	
6–12	5 (29%)
13–36	6 (35%)
37–60	4 (24%)
>60	2 (12%)

## 3 RESULTS

### 3.1 Themes and categories

The approach of thematic analysis by Braun and Clarke (2006) helped to extract meanings from the data and led to a group of important categories organized in three major themes: (a) physical environment, (b) social environment and (c) home‐like atmosphere. The themes provide a sense of the main concerns and perceptions of seniors living in ALFs. In each category, quotations are used to support findings from participants. To illustrate, the researcher referred to the first facility as ALF‐1, and to the second facility as ALF‐2. Table 2 provides a general summary of the themes and different dimensions in each theme.

**TABLE 2:** Themes and dimensions.

Theme #1: The physical environment	Theme #2: The social environment	Theme #3: Home‐like atmosphere
Physical space Design and accessibility Proximity to neighbourhood attractions Proximity to family Personal possessions Views and outdoor spaces	Relationship with residents Relationship with former friends Relationship with family Relationship with staff Community integration and activities	Meaning of home Food and dining Autonomy Privacy Independence

### 3.2 Physical environment

Participants valued physical environment differently and discussed various physical attributes such as physical space, design features, window views, outdoor spaces, proximity to neighbourhood attractions and proximity to family. Among these features, participants from both facilities considered physical space an important factor facilitating their movement and affecting their activities of daily living. As a whole, participants seemed to be most satisfied with their own personalized suites and personal possessions (e.g., furniture, books, pictures and decorative items). Many participants agreed that their personalized suites provided them with a home‐like atmosphere. However, participants with relatively shorter durations of living in the ALF seemed to be less attached to the facility, which made it hard for them to consider it as a home. Participants from both facilities lacked window views and outdoor spaces; they compared these features with what they had before at home. One of the main concerns for most participants was the location of the facility; both facilities were located in a place that lacked neighbourhood attractions, sidewalks and accessible public transportation. Therefore, improved physical access according to the physical needs of the residents can enhance the residents' experience of living in an ALF and improve their adaptation.

Researcher What was your initial thought about the place when you came in here for the first time?

Ben The room was not very large but it was sufficient.

Researcher You have mentioned that the room wasn't really large, how did you feel about that first?

Ben I felt it was very small compared with the house that I was moving out of; it was quite a change to try and put all our furnishings and things into a small apartment from a large house. (ALF‐2)

Researcher So what do you like most about this place?

Tim It's rather big and spacious with a kitchen in it so that we can make meals if you choose not to go to the dining room. And it's…when we came in and looked at the bedroom, ‘Oh you'll never get a king size bed in there’, well, we put a king size bed in there, it worked very nice thank you very much. It's worked out very comfortably ever since. (ALF‐1)

Researcher: Have you missed things you’ve been doing at home (e.g., your daily activities, hobbies, your own practices)? David Have you missed things you've been doing at home (e.g. your daily activities, hobbies, your own practices)?

David I would say, yes. The fact that I…my biggest entertainment back at home was getting to the senior's center down town‐ it was only five minutes away‐ and the activities that went on there.

Researcher So what do you like least about this place?

David It's location I think, I wish it was downtown. It's…other than that, that's the only thing‐ we're too far away in the bush I would say (ha, ha). (ALF‐1)

### 3.3 Social environment

Family and friends were among the most important people in participants' lives. Specifically, participants signified the role of their families and friends in supporting their transition and adjustment to the new facility. Most of the participants from both facilities appreciated the social atmosphere of the facility as an opportunity to socialize with other residents and staff, rather than living alone at home. After moving to the facility, few participants reported that they felt lonely and they expected many visits from family and friends. Few participants expressed challenges in forming relationships with others and preferred to stay alone in their rooms, especially at the beginning of their stay at the ALF. However, the majority seemed to be engaged in relationships with other residents. Participants who had frequent visits and contact with friends, children and other family members reported positive attitudes towards the facility and expressed more satisfaction. Participants repeatedly described how close relationships with loved ones (e.g. children, spouse, relatives and close friends) positively impacted their social life and helped them escape loneliness.

Researcher So what was your quality of life before moving here?

Paula I really didn't have a life. Like I said, I was alone, my children busy in their work and raising their little ones, so I really didn't have any quality of life at being alone at home; where I feel alive here.

Researcher So you say that you feel alive here, so how would you describe your quality of life now?

Paula Well in comparison you know, it's this way. I do feel quality, I feel at home, I feel friendship, I feel my social needs are met, my medical needs are taken care of, so it's good, it's good.

Researcher Have you changed doing things after moving in to this place?

Paula I am more involved than I was at home. At home I kind of wasted my time; I was depressed living alone and where here, like I say, I'm more involved. (ALF‐1)

Researcher In terms of your day to day life and living, have you changed the way that you do things, like your own practices, is it different from what you've been doing back home?

Eva Oh yes.

Researcher Can you talk about that?

Eva While I was living at home my family were nearby and they came often; I miss that‐they come, but not as often as they used to, there have been a lot of changes you just have to adjust to. (ALF‐1)

### 3.4 Home‐like atmosphere

In discussing the ALFs' home‐like features, participants emphasized physical features, social relationships, activities, care and services, food and dining, autonomy, privacy, independence, choice and security and safety. Participants compared the ALF to their own homes and they agreed that the facility became their current home. Different attributes contributed to participants' feeling at home. Participants rated these attributes differently; while some considered independence and choices as the main attributes that made them feel at home, others mentioned safety and security as major aspects that enhanced their sense of being at home.

Most participants indicated that their privacy was highly promoted inside the facility. However, two participants felt that the privacy of all residents was not highly respected. Most participants agreed that they had enough choices and freedom in their daily living such as mealtime, going and coming, recreation and activities. However, some participants complained about a lack of choices in their meal menu compared to their meals at their own homes. A major loss of independence for most of participants was the lack of ability to get somewhere without having to rely on others. Despite the fact that participants did not want to be dependent on others, even their family members, they found that the ALF was the best place to provide them assistance with daily living activities and health‐related needs given the physical and medical conditions they faced.

Researcher So if someone were to ask you to describe this place what words would you use to describe?

Paula Home; ya, its home to me. Some of the patients or the residents here can't say that. I don't know if it's because I'm more of a social person, I'm more independent, I don't know what it is, but I fit, I get along with everybody, and it's home. (ALF‐1)

Researcher So what does this place mean to you?

Gloria Well its home now.

Researcher Is it a typical home just like the home you left?

Gloria No.

Researcher Can you explain some differences?

Gloria Well we lived in our home for over thirty years here in town, and so living in the same place for that long you know, meant a lot. So I found it hard to leave. (ALF‐1)

## 4 DISCUSSION

### 4.1 Physical environment

The physical environment was discussed by residents in relation to many aspects of daily living. The existing literature pertained to several features of physical environment of ALFs. Suitably designed and maintained physical features of ALFs can help improve residents' perception of quality of life. Furthermore, environmental manipulations that increase resident comfort and privacy may improve resident satisfaction and well‐being (Chaudhury et al., 2018). Physical attributes of the facility positively impacted residents' feelings of being at home. Yet, some residents were dissatisfied with many features (e.g. physical space, lack of neighbourhood attractions, room configuration and design features).

The various perceptions of the physical environment stem from residents' personal expectations, compared to their former living conditions and their physical and functional abilities. Freedman et al. (2017) linked housing satisfaction to successful aging. They found that seniors who lived in accessible environments perceived their residence as being meaningful and useful. When the housing environment maximized residents' independence and autonomy, seniors reported a better sense of well‐being (Freedman et al., 2017). Findings from this study highlighted a need to confirm a relationship between physical conditions of housing and subjective sense of well‐being among seniors.

### 4.2 Social environment

Family and friends played a significant role in supporting residents' transitions to the ALF and adjustments to the new atmosphere. Attitudes and behaviours of staff seemed to significantly impact residents' sense of being at home. Paudel et al. (2021) found that positive relationships with staff were significant for improved quality of life in ALFs. In this study, stories presented by residents revealed the social aspect of care and how interaction with staff affected their lives. Residents labelled relationships with staff and different aspects of care provided to them as ‘very good,’ ‘excellent’ and ‘nice’. The main complaint from most residents was about a staff shortage.

The two facilities have created community activities for residents; from participants' points of view, most of these activities reflected facility programs, which may or may not reflect residents' needs and preferences. Moreover, many factors seemed to comprise residents' ability to interact with the larger community. For example, the facility must know when the resident is leaving and returning, many residents lacked access to transportation and those with a physical disability expressed a need for physical assistance outside the facility. These findings are largely congruent with the existing literature. Kane et al. (2005) argued that residential care settings tend to resemble community status to the extent that residents can access the greater community for activities of interest. Activities must be appropriate for residents' age and match their social profile, including education, employment, and recreation. Park (2009) suggested that ALFs could promote residents' psychological well‐being by encouraging them to develop meaningful relationships. Findings from this study revealed that integration in the assisted living community had two potential consequences – benefit or hindrance. It could help residents become involved in a supportive assisted living community to compensate for the lost relationships with the greater community. Conversely, the assisted living community may create more tension and controversy in interpersonal relationships among residents, especially for those who are not in alignment with other residents, and who are not interested in the social programs offered by the facility.

### 4.3 Home‐Like atmosphere

Despite offering a home‐like atmosphere, a move from home to an ALF remained a significant transition and major life alteration in the lives of residents. Adjustment to an assisted living life remained a difficult process. In this study, the researcher was able to capture diverse views on residents' sense of being home at the facility. The main philosophy of ALFs is to foster home‐like features or atmosphere that enhances this sense. However, the philosophy as stated by the facility is often different from what is actually offered to residents and what is understood in relation to the residents' own philosophies and reported experiences. This study revealed a range of feelings and perceptions about the importance of creating a home‐like atmosphere in such facilities.

For seniors, the meaning of home is an important factor that affects the process of relocation to another place of residence; it signifies the consequence of adjustment to a new environment. For participants, different patterns in the meaning of home reflected differences in values, needs, moving decisions and coping mechanisms. I encouraged participants to share descriptions of the meaning of home.

Paula referred to ALF‐1 as her home. She rationalized her sense of being at home to the sense of independence she felt, her tendency towards socializing and her adjustment and adaptation.

Food and dining were regarded as important factors contributing to residents' quality of life in many ways. Meal schedules and menu options affected the residents' autonomy and appeared frequently in residents' talk. Many residents expressed dissatisfaction with their limited food choices and the strict meal times. While most residents found themselves adjusting to mealtimes and food options after a period of residency, some did not. These residents felt restricted by being on a diet and the limited menu options. Most residents valued the dining table and enjoyed socializing with other residents during meal times. However, residents preferred home‐cooked food to institutional food, given the differences in food preparation and seasoning methods. Similar findings were reported by Frankowski et al. (2011), who intensively interviewed residents in ALFs. Frankowski et al. observed how the regular meal times and the predictive nature of the dining room helped incoming residents to adjust successfully to their new living space.

Assisted living facilities are distinguished from other residential care settings by providing a home‐like environment that maximizes autonomy, independence, and privacy. However, maximizing the independence of physically disabled and cognitively impaired residents remains a challenge (Hawes & Kimbell, 2010; Government of Alberta, 2014). Bramley et al. (2020) found that older people perceive their ability to maintain independence, autonomy, and individuality as the most important criterion for determining quality of life. Lee et al. (2009) discussed how seniors expressed varying levels of autonomy, perceptions of self‐sufficiency, and independence, which are highly affected by residents' personal profiles and coping mechanisms. Similarly, Robison et al. (2011) found that seniors' experiences of privacy, autonomy and dignity in a residential care setting are influenced by residents' personal characteristics such as age, perceived health, involvement in the decision to move to the residence and length of stay in the residence. Robison et al. (2011) also assessed the perceived quality of life in relation to many indicators, including autonomy, privacy, and independence. Among the different residential settings, assisted living residents reported the highest levels of privacy and autonomy.

### 4.4 Limitations of the study

Access to senior homes and their residents requires lots of coordination, permissions and ethical considerations. Researchers have faced delays in data collection due to facility‐related issues and the fact that seniors are vulnerable population. Both ALFs lacked diversity in terms of ethnic and cultural background, which limited the study's ability to explore variations in older populations. Qualitative research findings may reflect the researcher's perspective and interpretation; different readers may draw different conclusions from the same data, leading to potential subjectivity in the research's interpretation.

This study was also limited by being conducted in two ALFs in one geographic region. Future studies may consider the involvement of ALFs from different geographic regions across the country to enhance the applicability and transferability of the findings. Interviews were conducted with residents who were cognitively intact. Residents diagnosed with dementia and Alzheimer's disease were excluded given the cognitive problems that may affect the reliability of the study. Therefore, a further limitation is the lack of perceptions on how cognitively impaired residents might describe their own quality of life.

## 5 CONCLUSION

In this study, we explored seniors' perspectives on quality of life in assisted living facilities (ALFs) to gain a comprehensive understanding of this concept in the ALF context. Quality of life in ALFs is primarily influenced by the alignment between residents' adaptability and the facility's ability to meet their diverse needs. Our findings emphasize that quality of life is a highly individualized concept, varying among residents.

This research highlights the critical factors to consider when assessing the quality of life for seniors in ALFs. Resident satisfaction hinges on meeting their unique needs and expectations, influenced by a complex interplay of dynamic factors that extend beyond the facility's offerings. Quality of life can also be influenced by residents' adaptability and adjustment to their environment. Therefore, involving experts from diverse disciplines is crucial to understanding the variations among residents. Engaging residents and their families in decision‐making, soliciting their perceptions of quality of life before and after residing in an ALF, is essential for comprehending how they prioritize different aspects of their lives.

### 5.1 Implications for research

The existing body of literature gives limited attention to quality of life in ALFs and there is little research examining the views and voices of seniors who reside in these settings. It is important to continue to strive to understand quality of life for senior people living in ALFs. Taking into account all aspects identified by seniors themselves will be the key to achieving a successful ALF that recognizes quality as a multifaceted concept. The findings also demonstrated the need for further research that considers quality as a dynamic process over time. This is especially important for seniors who experience many physical and mental changes as they age and therefore, express dynamic and various needs. Furthermore, it is vital to gather staff perspectives on seniors' quality of life, both before and during their employment at ALFs. Incorporating input from all stakeholders will help identify areas of improvement and facilitate the development of solutions to enhance the quality of life for senior residents in ALFs.

### 5.2 Implications for practice

An important recommendation for health professionals, policymakers and administrators is to find a dynamic balance that considers the relationship between residents' personal needs and expectations, and what the facility can actually offer. Residents' personal needs should be embraced at all stages of transition to ALFs. Organizations are encouraged to regularly assess the personal needs and expectations of the residents upon their admission and throughout the duration of their stay. Organizations should ensure that an advocate is provided for each resident as and when needed. In addition, it is important to apply a process for continuous quality improvement/quality assurance and ensure that all residents and their family members are involved in the process.

## FUNDING INFORMATION

The study was funded by Covenant Health Research Centre, AB, Canada. Study#1441; Pro00039427 Approval obtained July 2013.

## CONFLICT OF INTEREST STATEMENT

The authors declare no conflict of interest for this article.

## ETHICS STATEMENT

The study was approved by the Covenant Health Research Centre and the Health Research Ethics Board, University of Alberta. Date of approval: 19 April 2013 Study ID: Pro00039427.

## Data Availability

The data that support the findings of this study are openly available in university publications.

## APPENDIX 1 Resident interview protocol

**Project Title: ‘Seniors’ Experiences in a Designated Assisted Living Facility'**

**Date:** _____________________________________

**Pseudonym:** _____________________________________

**Table:**

**Gender:**	○Male	○Female
**Age:**	65 and older	

**Marital Status:**

**Education:**

**Occupation:**

**Location of primary residence prior to residing at DAL Facility:**

**Introduction**

The research study will explore experiences of life among residents living in a designated assisted living facility located in Southern Alberta.

**Purpose**

The purpose of my research is to explore the experiences of life among seniors living at this designated assisted living facility. The researcher is interested in learning about what your life is like living at this facility and what matters most to you in terms of quality of life. This information will assist me in developing insights into ‘quality of life’ as perceived by residents themselves.

**Consent Form**

Before we start I would like to go through the consent form with you.

[*Read through the consent form with them*; *ask if they have any questions*; *do they understand the consent form*; *if they are okay with things*, *have them to sign off on the consent which will indicate their agreement to participate in the research*.]

**Audio‐Recorder:** As I mentioned earlier, I would like to audio record this interview, upon your consent, to ensure accuracy in the transcription of the data. Do you consent to have the interview recorded? [*Ensure their consent*; *if consent is provided*, *have them sign off on this on the consent form*.]
**Review of Transcription:** I plan to have this interview transcribed in a 4‐week period; do you anticipate that you would like to see a copy of the transcribed interview and to make any necessary changes to the provided transcription prior to it being included in my analysis? [*If they would like to see a copy of the transcribed interview*, *have them check this off on the consent form*, *then you will have to contact them in 4 weeks to allow them to review the document*.]
**Questions:** Now before we start, do you have any questions that I could answer for you at this time?

[*Turn on the recording device*.] Participants will be notified that recording starts at this point.

**Interview Questions**

1. **The Facility**
  - How long have you been living at this facility?
  - Prior to moving to this facility, did you look into a few options as far as assisted living facilities? Please elaborate.
  - How did you come to know about this facility?
  - What attracted you to this facility?
  - Were you looking forward to this move?
  - What were your initial impressions when you moved into this facility?
  - How long did it take you to adjust to living here?
  - What is it like living here at this place? [Tell me about your experience of living at this facility … I want to get a better idea of life here at this facility.]
  - What is a typical day like living in this place? [Have them describe a typical day.]
  - After living here for a period of time now, what do you feel is unique about living here at this facility?
2. **Values**
  - In terms of your day to day life and living, what is important to you … what things are important to you? [Are you able to practice these things, e.g. praying, meditation, playing music…]
  - Is what was important to you before you moved here still important? Please explain.
3. **Meaning**
  - If someone were to ask you to describe this place, what words would you use to describe?
  - What do you like most about this place? What kinds of things here do you enjoy?
  - What do you like least about this place? What do you not enjoy about this place?
  - Are you happy here?
  - Have you met people here that you would call friends?
  - What does this place mean to you?
4. **Quality**
  - What was your quality of life like before moving to this facility? How would you describe your life before?
  - How would you describe your quality of life now living here? Has it changed? How so?
  - How would you describe the quality of service that you receive from the staff here?
  - Has something happened over time that has changed your perception of this …, e.g. an incident that may have influenced your definition of quality of life while living here at this facility? [Can they provide a specific(s) example?]
  - Would you recommend this facility to a family member or friend?
5. **Challenges**
  - What are some of the challenges to your quality of life that present living in a facility such as this?
  - How do you cope with or manage these challenges?
6. **Autonomy**
  - Do people in this facility allow you to choose whatever you want? [What are some examples of things that you can choose or decide about it? What are some examples of things that you cannot choose or decide about it?]
  - Do you think that this facility respects your personal choices? How?
7. **Change**
  - Have you changed the way that you do things at this facility? [Probe: activities of daily living (e.g. eating habits, bed time), communication with family and friends, leisure and recreation activities, hobbies …] Are you comfortable with these changes?
  - Can you give an example of when a change in the way of doing things was necessary? How has this affected you and your day‐to‐day life?
  - What are some changes would you like to see in this place that might improve your life quality?
8. **Other**
  - Before we conclude the interview, is there anything you would like to add … any additional comments?

[*Thank the interviewee for their time and for participating in the study*.]

[*Turn off recording device*.]
